# Phylogeny and diversity of *Haploporus* (Polyporaceae, Basidiomycota)

**DOI:** 10.3897/mycokeys.54.34362

**Published:** 2019-06-12

**Authors:** Meng Zhou, Li Wang, Tom W. May, Josef Vlasák, Jia-Jia Chen, Yu-Cheng Dai

**Affiliations:** 1 Beijing advanced innovation centre for tree breeding by molecular design, Institute of Microbiology, PO Box 61, Beijing Forestry University, Beijing 100083, China; 2 School of Economics and Management, Beijing Forestry University, Beijing 100083, China; 3 Royal Botanic Gardens Victoria, Melbourne, Victoria 3004, Australia; 4 Biology Centre of the Academy of Sciences of the Czech Republic, Branišovská 31, CZ-370 05 České Budějovice, Czech Republic; 5 College of Plant Protection, Nanjing Agricultural University, Nanjing, Jiangsu 210095, China; 6 Beijing Advanced Innovation Center for Tree Breeding by Molecular Design, Beijing Forestry University, Beijing 100083, China

**Keywords:** Polyporales, taxonomy, wood-inhabiting fungi

## Abstract

Four species of *Haploporus*, *H.angustisporus*, *H.crassus*, *H.gilbertsonii* and *H.microsporus* are described as new and *H.pirongia* is proposed as a new combination, based on morphological characteristics and molecular phylogenetic analyses inferred from internal transcribed spacer (ITS) and large subunit nuclear ribosomal RNA gene (nLSU) sequences. *Haploporusangustisporus*, *H.crassus* and *H.microsporus* occur in China, *H.gilbertsonii* occurs in the USA, and the distribution of *H.pirongia* is extended from New Zealand to Australia. *Haploporusangustisporus* is characterized by the distinct narrow oblong basidiospores measuring 10.5–13.5 × 3.9–5 µm. *Haploporuscrassus* is characterized by the presence of ventricose cystidioles occasionally with a simple septum, dissepimental hyphae usually with a simple septum, unique thick-walled basidia and distinctly wide oblong basidiospores measuring 13.5–16.5 × 7.5–9.5 µm. *Haploporusgilbertsonii* is characterized by its large pores (2–3 per mm), a dimitic hyphal structure with non-dextrinoid skeletal hyphae and wide oblong basidiospores measuring 12–15 × 6–8 µm. *Haploporusmicrosporus* is characterized by distinctly small pores (7–9 per mm), the presence of dendrohyphidia, and distinctly small ellipsoid basidiospores measuring 5.3–6.7 × 3–4.1 µm. *Haploporuspirongia* is proposed as a new combination. *Haploporusamarus* is shown to be a synonym of *H.odorus* and *Pachykytosporawasseri* is considered a synonym of *H.subtrameteus*.

## Introduction

*Haploporus* Bondartsev & Singer (Polyporales, Basidiomycota) is characterized by annual to perennial, resupinate to pileate basidiocarps, a di- to trimitic hyphal system with clamped connections on the generative hyphae, cyanophilous skeletal hyphae, cylindrical to subglobose, hyaline, thick-walled, cyanophilous and ornamented basidiospores, and formation of a white rot (Singer 1944, Dai et al. 2002, Piątek 2005, Li et al. 2007, Shen et al. 2016). *Pachykytospora* was shown to be, micro-morphologically, similar to Haploporus, differing only in having resupinate basidiocarps; both names were treated as synonyms (Dai et al. 2002) and consequently, all Pachykytospora species have been transferred to Haploporus (Dai et al. 2002, Piątek 2005, Shen et al. 2016), but *P.major* G.Y.Zheng&Z.S.Bi (add lit.), which belong to Megasporia because of its thin-walled and smooth basidiospores (Dai and Li 2002). The monophyly of Pachykytospora was confirmed later on by molecular analysis (Shen et al. 2016). So far 13 species have been accepted in *Haploporus* (Dai et al. 2002, Hattori et al. 2002, Piątek 2005, Li et al. 2007, Dai and Kashiwadani 2009, Shen et al. 2016).

During a study on taxonomy of Polyporaceae, several specimens of *Haploporus* from USA, Australia and China were studied. After morphological examinations and phylogenetic analysis of ITS and nLSU sequences, four new species were confirmed to be members of the *Haploporus* lineage. In this paper, we describe and illustrate these new species. In addition, *Poriapirongia* G. Cunn. was originally described from New Zealand (Cunningham 1947), and treated as a synonym of *Poriapapyracea* (Schwein.) Cooke (= *Haploporuspapyraceus* (Schwein.) Y.C.Dai&Niemelä (Cunningham 1965, Lowe 1966 and Buchanan and Ryvarden 1988) is shown to represent an independent species, based on new specimens and both morphology and phylogenetic evidences. Therefore, a new combination (*H.pirongia*) is proposed.

## Materials and methods

### Morphological studies

Sections were studied microscopically according to Dai (2010) at magnifications ≤1000× using a Nikon Eclipse 80i microscope with phase contrast illumination. Drawings were made with the aid of a drawing tube. Microscopic features, measurements, and drawings were made from sections stained with Cotton Blue and Melzer’s reagent. Spores were measured from sections cut from the tubes. To present spore size variation, the 5% of measurements excluded from each end of the range are given in parentheses. Basidiospore spine lengths were not included in the measurements. Abbreviations include: IKI = Melzer’s reagent, IKI– = negative in Melzer’s reagent, KOH = 5% potassium hydroxide, CB = Cotton Blue, CB+ = cyanophilous, L = mean spore length (arithmetic average of all spores), W = mean spore width (arithmetic average of all spores), Q = the L/W ratio, and n = number of spores measured / from given number of specimens. Color terms follow Petersen (1996). Herbarium abbreviations follow Thiers (2018).

### Molecular study and phylogenetic analysis

A CTAB rapid plant genome extraction kit (Aidlab Biotechnologies, Beijing) was used to obtain PCR products from dried specimens, according to the manufacturer’s instructions with some modifications (Cao et al. 2012, Zhao et al. 2013). The DNA was amplified with the primers: ITS5 and ITS4 for ITS (White et al. 1990), and LR0R and LR7 (http://www.biology.duke.edu/fungi/mycolab/primers.htm) for nLSU (Vilgalys and Hester 1990). The PCR procedure for ITS was as follows: initial denaturation at 95 °C for 3 min, followed by 34 cycles at 94 °C for 40 s, 54 °C for 45 s and 72 °C for 1 min, and a final extension of 72 °C for 10 min. The PCR procedure for nLSU was as follows: initial denaturation at 94 °C for 1 min, followed by 34 cycles at 94 °C for 30 s, 50 °C for 1 min and 72 °C for 1.5 min, and a final extension of 72 °C for 10 min. The PCR products were purified and sequenced at the Beijing Genomics Institute, China with the same primers.

*Phylogenetic analyses*. New sequences, deposited in GenBank (http://www.ncbi.nlm.nih.gov/genbank/) (Table 1), were aligned with additional sequences retrieved from GenBank (Table 1) using BioEdit 7.0.5.3 (Hall 1999) and ClustalX 1.83 (Thompson et al. 1997). The sequence quality were checked followed Nilsson et al. (2012). *Perenniporiahainaniana* B.K.Cui&C.L.Zhao and *P.medulla-panis* (Jacq.) Donk were used as outgroups, following Shen et al. (2016). Prior to phylogenetic analysis, ambiguous regions at the start and the end of the alignment were trimmed and gaps were manually adjusted to optimize the alignment were trimmed. The edited alignment was deposited at TreeBase (http://purl.org/phylo/treebase; submission ID 24089).

**Table 1.** Information on the sequences used in this study.

Species	Sample no.	Location	GenBank accession no.	
			ITS	nLSU
* Haploporus alabamae *	JV_0610_K16-Kout	Belize	KY264039	
	Dollinger 895	USA	KY264038	**MK433606**
	JV 1704/75	Costa Rica	MK429754	**MK433607**
*** H. angustisporus ***	**Cui 9046**	**China**	**KU941862**	**KU941887**
	**Dai 10951**	**China**	**KX900634**	**KX900681**
*** H. crassus ***	**Dai 13580**	**China**	**FJ627252**	**KU941886**
* H. cylindrosporus *	Dai 15643	China	KU941853	KU941877
	Dai 15664	China	KU941854	KU941878
*** H. gilbertsonii ***	**JV 1209/63-J**	**USA**	**MK429755**	**MK433608**
	**JV 1611/5-J**	**USA**	**MK429756**	**MK433609**
* H. latisporus *	Dai 11873	China	KU941847	KU941871
	Dai 10562	China	KU941848	KU941872
*** H. microsporus ***	**Dai 12147**	**China**	**KU941861**	**KU941885**
* H. nanosporus *	LYAD 2044a	Gabon	KU941859	KU941883
	LYAD 2044b	Gabon	KU941860	KU941884
* H. nepalensis *	Dai 12937	China	KU941855	KU941879
	Cui 10729	China	KU941856	KU941880
* H. odorus *	Dai 11296	China	KU941845	KU941869
	Yuan 2365	China	KU941846	KU941870
H. cf. odorus	KUC20121123-29	Republic of Korea	KJ668537	KJ668390
* H. papyraceus *	Dai 10778	China	KU941839	KU941863
	Cui 8706	China	KU941840	KU941864
	KUC20130719-04	Republic of Korea	KJ668535	KJ668388
*** H. pirongia ***	**Dai 18659**	**Australia**	**MH631017**	**MH631021**
	**Dai 18660**	**Australia**	**MH631018**	**MH631022**
	**Dai 18661**	**Australia**	**MH631019**	**MH631023**
	**Dai 18662**	**Australia**	**MH631020**	**MH631024**
	**PDD 95714**	**New Zealand**	**MK429757**	
* H. septatus *	Dai 13581	China	KU941843	KU941867
	Cui 4100	China	KU941844	KU941868
*H.* sp.	KUC20080606-35	Republic of Korea	KJ668534	KJ668387
* H. subpapyraceus *	Dai 9324	China	KU941841	KU941865
	Cui 2651	China	KU941842	KU941866
* H. subtrameteus *	Dai 4222	China	KU941849	KU941873
	Cui 10656	China	KU941850	KU941874
	Dai11270	China	KY264042	
H. cf. subtrameteus	KUC20121102-36	Republic of Korea	KJ668536	KJ668389
* H. thindii *	Cui 9373	China	KU941851	KU941875
	Cui 9682	China	KU941852	KU941876
* H. tuberculosus *	15559	Sweden	KU941857	KU941881
	15560	Austria	KU941858	KU941882
*H.tuberculosus* (as *Pachykytospora*)	KA11 (GB)	Sweden	JX124705	
	JV 9610/20	Slovakia	KY264040	**MK433610**
	JV 0509/19	Czech Republic	KY264041	**MK433611**
* Pachykytospora wasseri *	LE814872 (T)	Russia	KM411456	KM411472
* Perenniporia hainaniana *	Cui 6364	China	JQ861743	JQ861759
* P. medulla-panis *	Cui 3274	China	JN112792	JN112793

Maximum parsimony (MP) and Bayesian inference (BI) were employed to perform phylogenetic analysis of the two aligned datasets. The two phylogenetic analysis algorithms generated nearly identical topologies for each dataset, and, thus only the topology from the MP analysis is presented along with statistical values from the MP and BI algorithms. Most parsimonious phylogenies were inferred from the ITS + nLSU, and their combinability was evaluated with the incongruence length difference (ILD) test (Farris et al. 1994) implemented in PAUP* 4.0b10 (Swofford 2002), under a heuristic search and 1000 homogeneity replicates giving a P value of 1.000, much greater than 0.01, which means there is no discrepancy among the two loci in reconstructing phylogenetic trees. Phylogenetic analysis approaches followed Zhao et al. (2015). The tree construction procedure was performed in PAUP* version 4.0b10 (Swofford 2002). All characters were equally weighted, and gaps were treated as missing data. Trees were inferred using the heuristic search option with TBR branch swapping and 1000 random sequence additions. Max-trees were set to 5000, branches of zero length were collapsed and all parsimonious trees were saved. Clade robustness was assessed using a bootstrap (BT) analysis with 1000 replicates (Felsenstein 1985). Descriptive tree statistics tree length (TL), consistency index (CI), retention index (RI), rescaled consistency index (RC), and homoplasy index (HI) were calculated for each maximum parsimonious tree (MPT) generated. jModeltest v.2.17 (Darriba et al. 2012) was used to determine the best-fit evolution model of the combined dataset for Bayesian inference (BI). The Bayesian inference (BI) was conducted with MrBayes 3.2.6 (Ronquist et al. 2012) in two independent runs, each of which had four chains for 10 million generations and started from random trees. Trees were sampled every 1000^th^ generation. The first 25% of sampled trees were discarded as burn-in, whereas other trees were used to construct a 50 % majority consensus tree and for calculating Bayesian posterior probabilities (BPPs).

Phylogenetic trees were visualized using Treeview (Page 1996). Nodes that received Bootstrap support ≥50% and Bayesian posterior probabilities (BPP) ≥0.90 are considered as significantly supported.

## Results

### Molecular phylogeny

**Figure 1.**
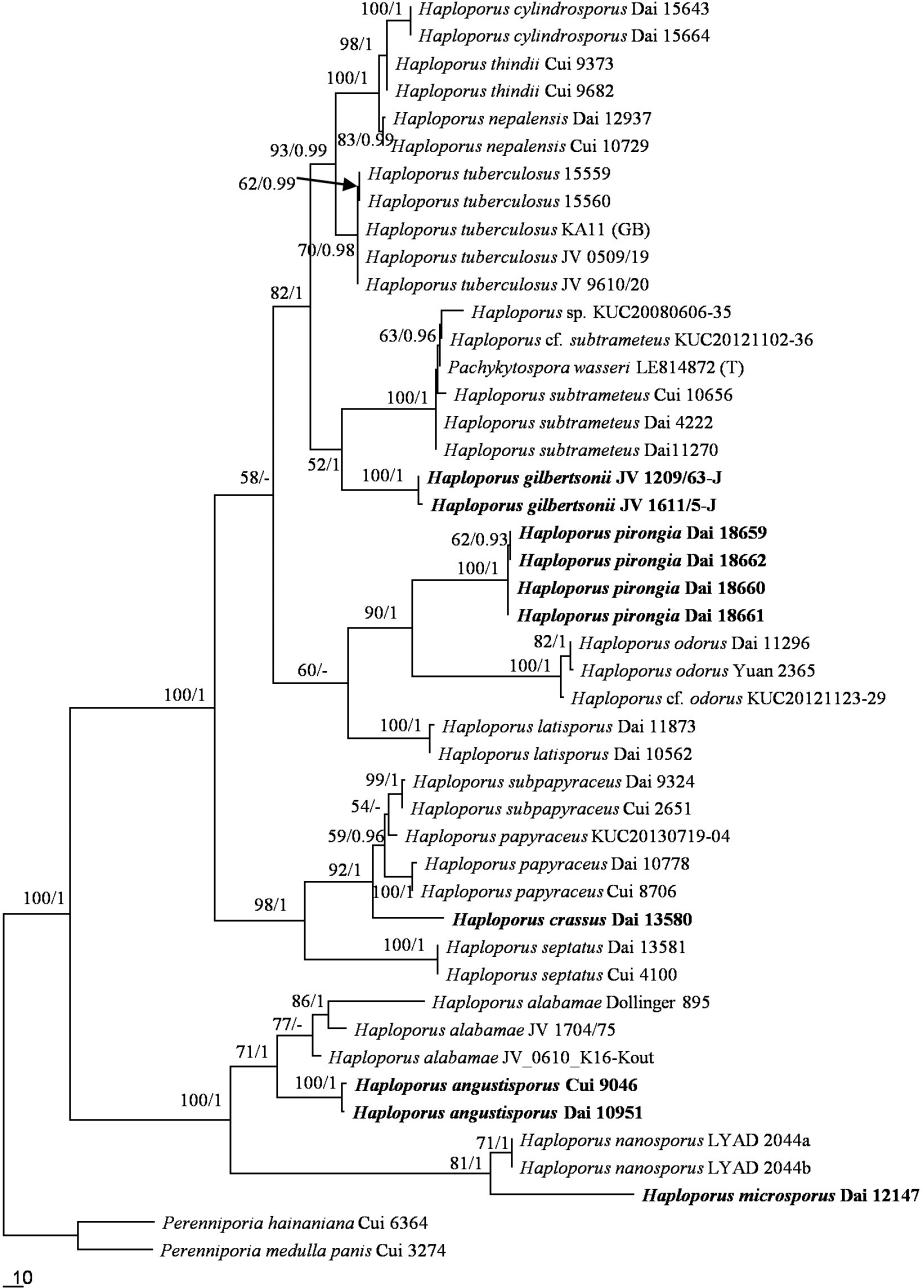
Maximum parsimony strict consensus tree illustrating the phylogeny of *Haploporus* based on ITS+nLSU sequences. Branches are labeled with parsimony bootstrap proportions (before slanting line) greater than 50% and bayesian posterior probabilities (after slanting line) greater than 0.90.

**Figure 2.**
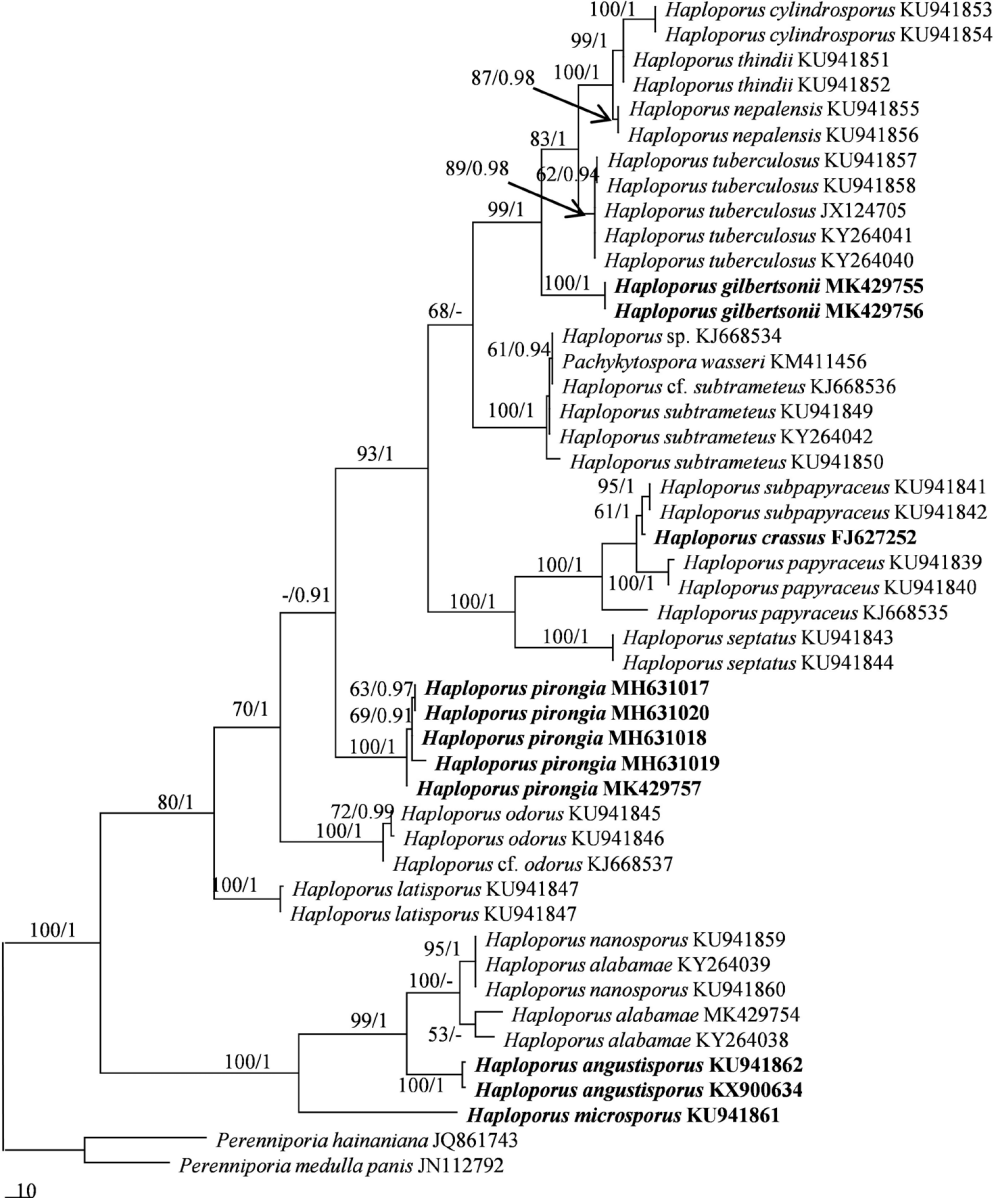
Maximum parsimony strict consensus tree illustrating the phylogeny of *Haploporus* based on ITS sequences. Branches are labeled with parsimony bootstrap proportions (before slanting line) greater than 50% and bayesian posterior probabilities (after slanting line) greater than 0.90.

The combined ITS and 28S dataset included sequences from 46 fungal collections representing 21 species. The dataset had an aligned length of 2054 characters, of which 1399 characters are constant, 98 are variable and parsimony-uninformative, and 557 are parsimony-informative. MP analysis yielded 4 equally parsimonious trees (TL = 1370, CI = 0. 639, RI = 0.870, RC = 0.556, HI = 0.361). The best model for the combined ITS and 28S sequences dataset estimated and applied in the BI was GTR+I+G. BI resulted in a similar topology with an average standard deviation of split frequencies = 0.004515 to MP analysis, and thus only the MP tree is provided. Both BT values (≥50%) and BPPs (≥0.90) are shown at the nodes (Fig. 1). The ITS-based phylogenies included ITS sequences from 47 fungal collections representing 21 species. The dataset had an aligned length of 711 characters, of which 317 characters are constant, 54 are variable and parsimony-uninformative, and 340 are parsimony-informative. MP analysis yielded 4 equally parsimonious trees (TL = 927, CI = 0. 653, RI = 0.888, RC = 0.580, HI = 0.347). The best model for the ITS sequences dataset estimated and applied in the BI was GTR+I+G. BI resulted in a similar topology with an average standard deviation of split frequencies = 0.005040 to MP analysis, and thus only the MP tree is provided. Both BT values (≥50%) and BPPs (≥0.90) are shown at the nodes (Fig. 2).

In both 28S+ITS- and ITS-based phylogenies (Figs 1–2), five new well-supported lineages were identified. Among them three well-supported terminal clades and two isolated branches (100% MP and 1.00 BI). *Haploporusangustisporus* is sister to *H.alabamae* (Berk. & Cooke) Y.C.Dai&Niemelä and this two species clade is related to *H.nanosporus* (A.David&Rajchenb.) Piątek, whereas *H.gilbertsonii* clustered with *H.cylindrosporus* L.L. Shen, Y.C.Dai&B.K.Cui, *H.thindii* (Natarajan & Koland.) Y.C.Dai, *H.nepalensis* (T. Hatt.) Piątek and *H.tuberculosus* (Fr.) Niemelä&Y.C.Dai. Four Australian specimens and a specimen of *Poriapirongia* from New Zealand formed a well-supported clade (100% MP and 1.00 BI), sister to the *H.odorus* clade. In addition, the other two lineages formed two distinct sublineages; *Haploporuscrassus* is closely related to *H.papyraceus* and *H.subpapyraceus* L.L.Shen, Y.C.Dai&B.K.Cui; whereas The *H.nanosporus* and *H.microsporus* clades are sister clades.

### Taxonomy

#### Haploporus angustisporus

Taxon classificationFungiPlagiorchiidaHaploporidae

Meng Zhou&Y.C.Dai sp. nov.

MB829583

Figs 3 , 4

##### Diagnosis.

Differs from other *Haploporus* species by the combination of its resupinate habit, a dimitic hyphal structure with dextrinoid skeletal hyphae, the absence of dendrohyphidia, and distinct narrow oblong basidiospores measuring 10–13.5 × 4–5 µm.

**Figure 3.**
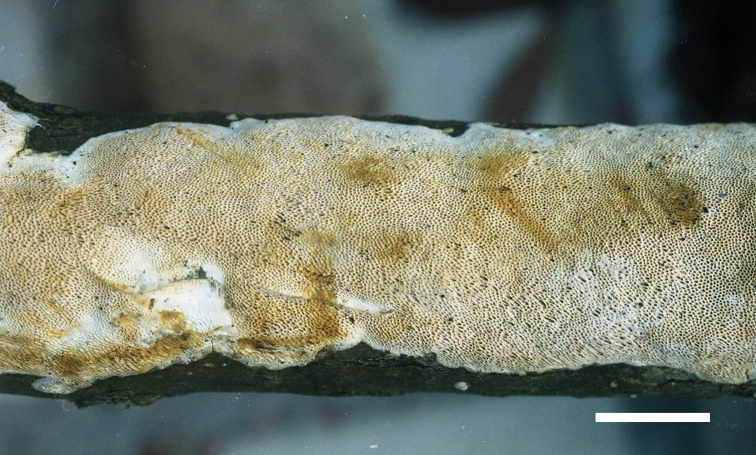
A basidiocarp of *Haploporusangustisporus* (Holotype). Scale bar: 1.0 cm.

**Figure 4.**
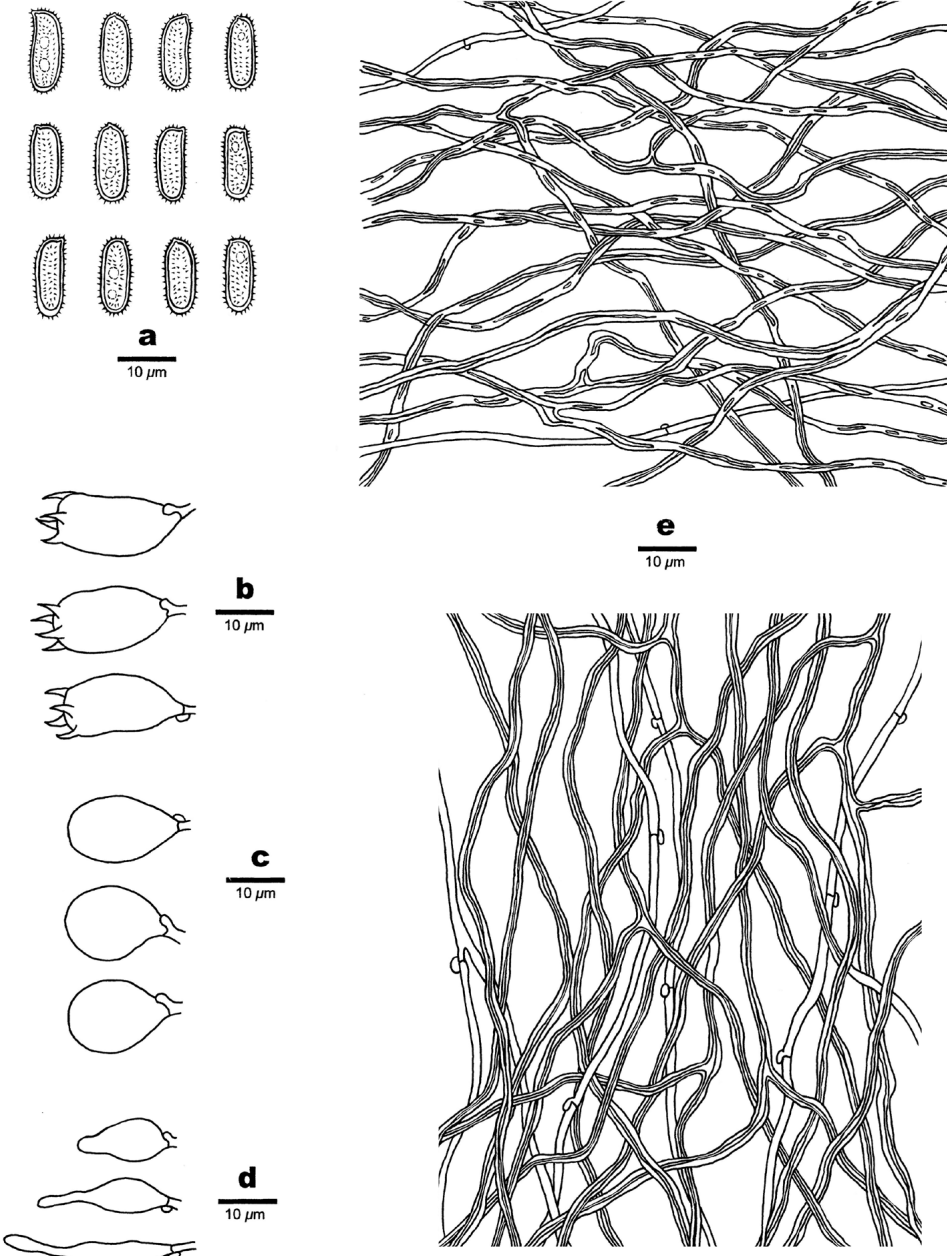
Microscopic structures of *Haploporusangustisporus* (Holotype). **a** Basidiospores **b** Basidia **c** Basidioles **d** Cystidioles **e** Hyphae from subiculum **f** Hyphae from trama.

##### Holotype.

CHINA. Guangdong Prov., Lianzhou County, Nanling Nat. Res., on fallen angiosperm branch, 15 May 2009, *Dai 10951* (Holotype in BJFC).

##### Etymology.

*Angustisporus* (Lat.): referring to the species having narrow basidiospores.

##### Fruitbody.

Basidiocarps annual, resupinate, adnate, soft corky when fresh, become corky upon drying, without odor or tasteless when fresh, up to 3 cm long, 2.5 cm wide, 2 mm thick at center. Pore surface cream to pale yellowish brown when fresh, brownish when bruised, olivaceous buff to pale brown upon drying; sterile margin indistinct, very narrow to almost lacking; pores angular, 3–5 per mm; dissepiments thick, entire. Subiculum cream, corky, thin, about 0.1 mm thick. Tubes light buff, corky, about 1.9 mm long.

##### Hyphal structure.

Hyphal system dimitic: generative hyphae bearing clamp connections, hyaline, thin-walled; skeletal hyphae dominant, thick-walled, frequently branched, dextrinoid, CB+, tissues unchanging in KOH.

##### Subiculum.

Generative hyphae infrequent, hyaline, thin-walled, rarely branched, 1.5–2.5 µm in diam; skeletal hyphae dominant, hyaline, thick-walled with a narrow lumen to subsolid, frequently branched, interwoven, 1–2.5 µm in diam.

##### Tubes.

Generative hyphae frequent, hyaline, thin-walled, occasionally branched, 1.5–2.5 µm in diam; skeletal hyphae distinctly thick-walled with a narrow to wide lumen, frequently branched, interwoven, 1.2–2.5 µm in diam. Cystidia absent; cystidioles present, fusiform, 23–35 × 4–7 µm. Basidioles dominant, pear-shaped to subglobose, basidia barrel-shaped with 4-sterigmata and a basal clamp connection, 21–26 × 8–11 µm; . Dendrohyphidia absent. Some irregular-shaped crystals present among tube tramal structures.

##### Spores.

Basidiospores oblong, hyaline, thick-walled, with short tuberculate ornamentation, IKI–, CB+, 10–13.5(–14) × (3.5–)4–5 µm, L = 11.25 µm, W = 4.44 µm, Q = 2.38–2.70 (n = 60/2).

##### Additional specimen examined (paratype).

CHINA. Guangdong Prov., Fengkai County, Heishiding Nat. Res., on fallen angiosperm branch, 1 July 2010, *Cui 9046* (in BJFC).

#### Haploporus crassus

Taxon classificationFungiPlagiorchiidaHaploporidae

Meng Zhou&Y.C.Dai sp. nov.

MB829584

Fig. 5

##### Diagnosis.

Diﬀers from other *Haploporus* species by the combination of a resupinate habit, a dimitic hyphal structure with non-dextrinoid skeletal hyphae, the presence of ventricose cystidioles occasionally with a simple septum, dissepimental hyphae usually with a simple septum, unique thick-walled basidia and distinct wide oblong basidiospores measuring 13.5–16.5 × 7.5–9.5 µm.

**Figure 5.**
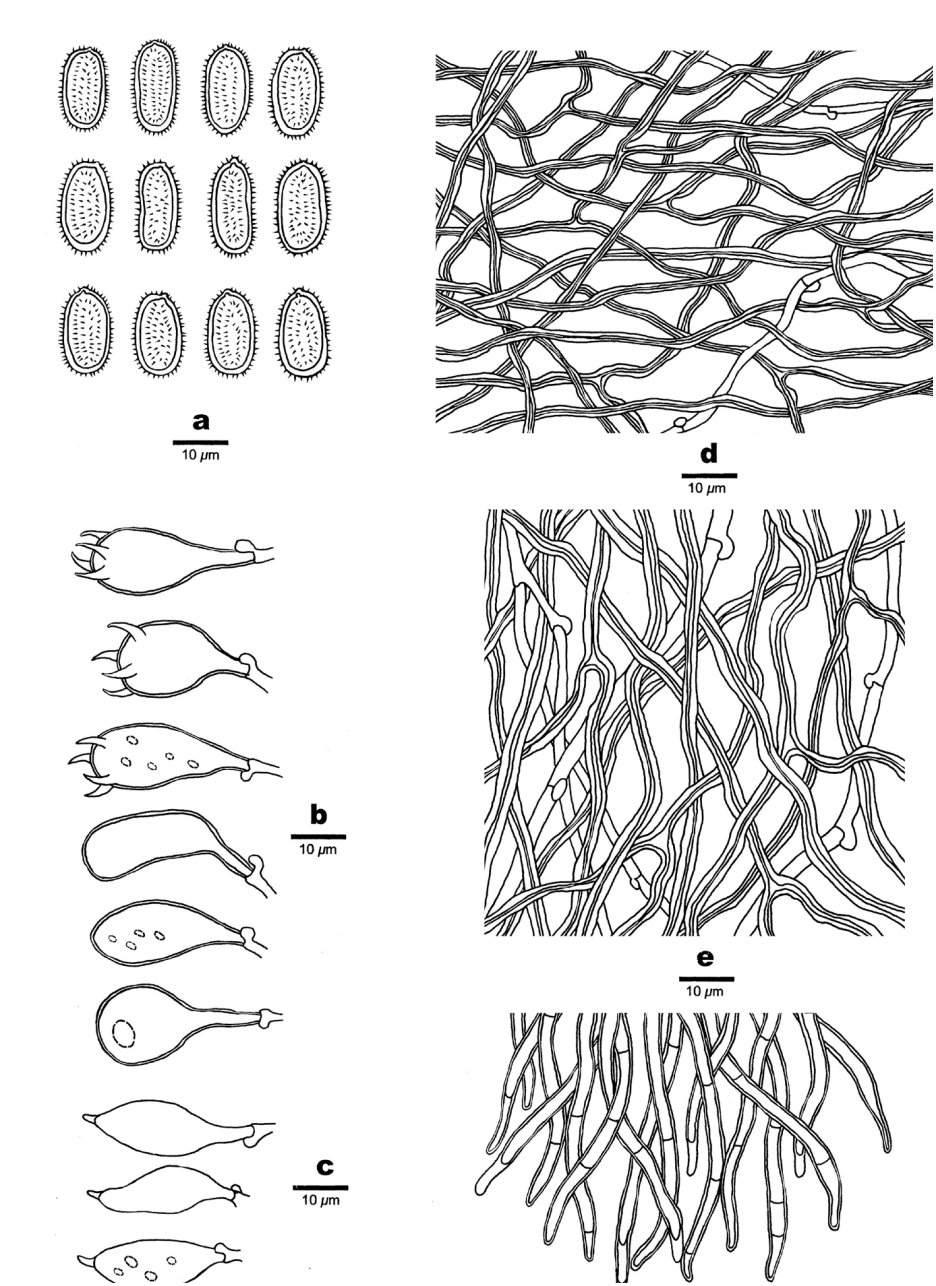
Microscopic structures of *Haploporuscrassus* (Holotype). **a** Basidiospores **b** Basidia and Basidioles **c** Cystidioles **d** Hyphae from subiculum **e** Hyphae from trama **f** Hyphae at dissepiment.

##### Holotype.

CHINA. Yunnan Prov., Xinping County, Ailaoshan Nat. Res., on rotten angiosperm wood, 15 Oct. 2013, *Dai 13580* (Holotype in BJFC).

##### Etymology.

*Crassus* (Lat.): referring to the species having wide basidiospores.

##### Fruitbody.

Basidiocarps annual, resupinate, adnate, soft corky when fresh, become corky and cracked upon drying, without odor or taste when fresh, up to 35 cm long, 3 cm wide and 1 mm thick at center. Pore surface white to cream when fresh, becoming buff-yellow upon drying; sterile margin indistinct, very narrow to almost lacking; pores round, 3–5 per mm; dissepiments thin, mostly entire, sometimes lacerate. Subiculum cream, corky, thin, about 0.1 mm thick. Tubes light buff, corky, about 0.9 mm long.

##### Hyphal structure.

Hyphal system dimitic: generative hyphae bearing clamp connections, hyaline, thin-walled; skeletal hyphae dominant, thick-walled, frequently branched, IKI–, CB+, tissues unchanging in KOH.

##### Subiculum.

Generative hyphae infrequent hyaline, thin-walled, rarely branched, 1.5–2.5 µm in diam; skeletal hyphae dominant, hyaline, thick-walled with a narrow lumen, frequently branched, interwoven, 1–2 µm in diam.

##### Tubes.

Generative hyphae frequent, hyaline, thin-walled, occasionally branched, 1.5–3 µm in diam; skeletal hyphae dominant, distinctly thick-walled with a narrow to wide lumen, frequently branched, interwoven, 1.5–2.5 µm in diam; dissepimental hyphae usually with a simple septum. Cystidia absent; cystidioles present, ventricose, usually with a small umbo having a simple septum, occasionally with a few small guttules, 21–31× 8–10 µm. Basidioles thick-walled, dominant, similar in shape to basidia, but smaller; basidia thick-walled, pear-shaped to barrel-shaped with 4-sterigmata and a basal clamp connection, occasionally with some small guttules, 22–31 × 8–13 µm; dendrohyphidia absent. Some irregular-shaped crystals present among tube tramal stru ctures.

##### Spores.

Basidiospores oblong, hyaline, thick-walled, with tuberculate ornamentation, IKI–, CB+, 13.5–16.5(–17) × (7–)7.5–9.5 µm, L = 15.06 µm, W = 8.15 µm, Q = 1.85 (n = 30/1).

#### Haploporus gilbertsonii

Taxon classificationFungiPlagiorchiidaHaploporidae

Meng Zhou, Vlasák&Y.C.Dai sp. nov.

MB829649

Figs 6 , 7

##### Diagnosis.

Diﬀers from other *Haploporus* species by its relatively large pores, 2–3 per mm, a dimitic hyphal structure with non-dextrinoid skeletal hyphae, the absence of dendrohyphidia, and wide oblong basidiospores measuring 12–15 × 6–8 µm.

**Figure 6.**
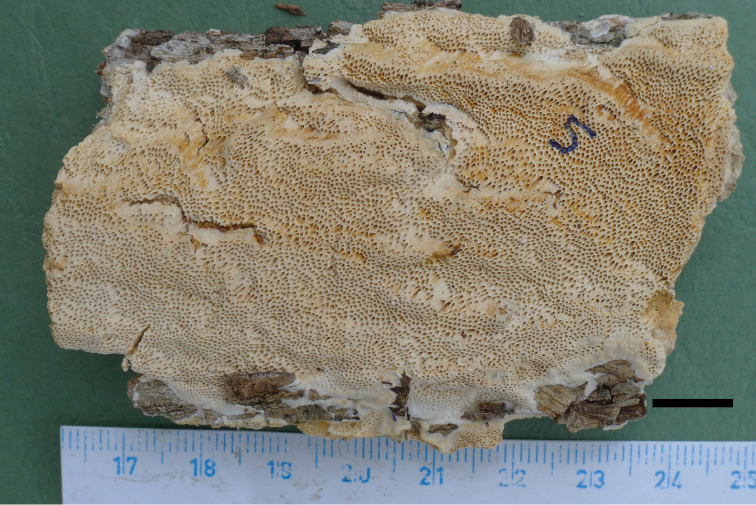
A basidiocarp of *Haploporusgilbertsonii* (Holotype). Scale bar: 1.0 cm.

**Figure 7.**
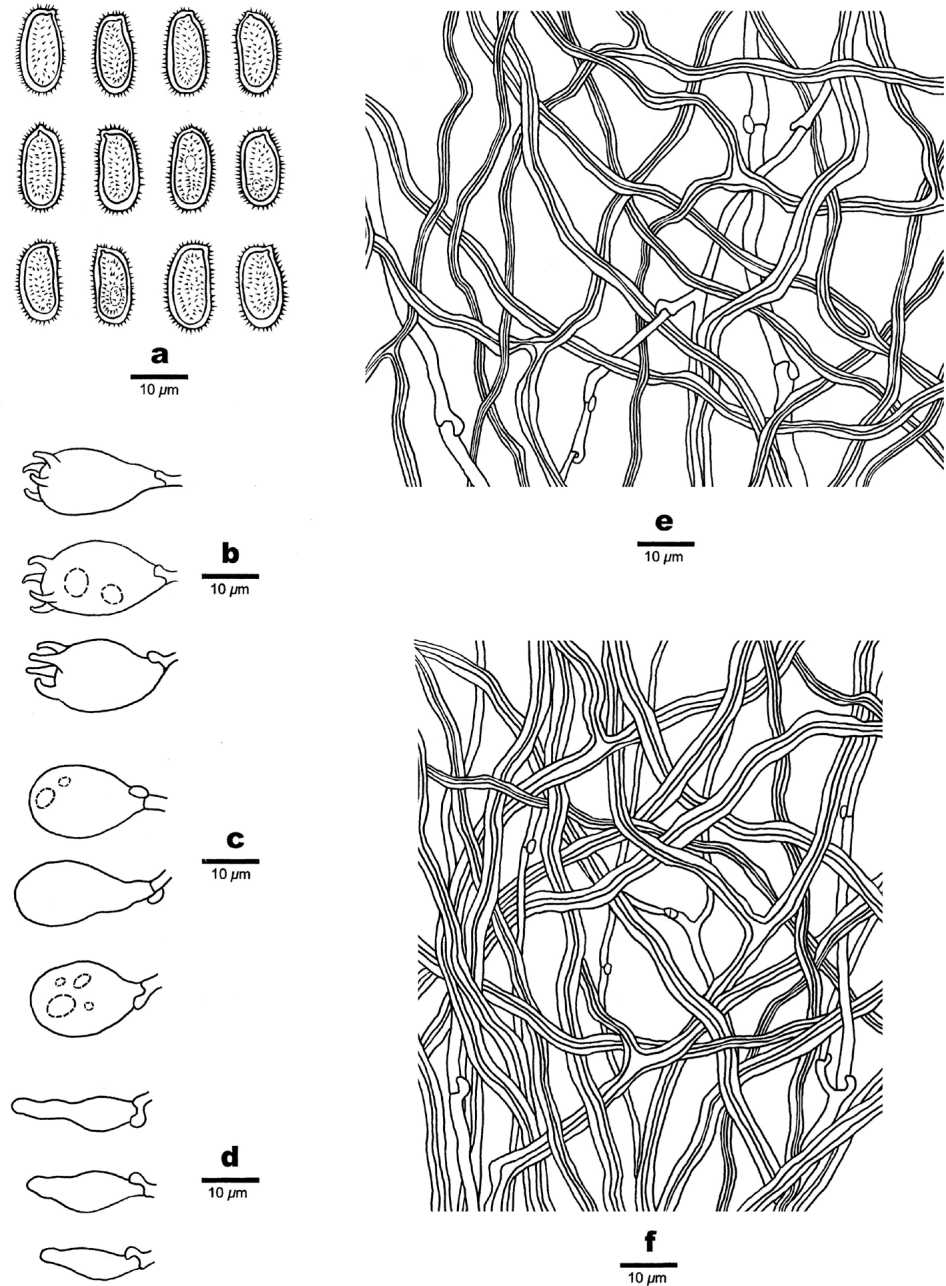
Microscopic structures of *Haploporusgilbertsonii* (Holotype). **a** Basidiospores **b** Basidia **c** Basidioles **d** Cystidioles **e** Hyphae from subiculum **f** Hyphae from trama.

##### Holotype.

USA. Arizona, Santa Rita Mt., Madera Canyon, on dead tree of *Quercus*, 20 Nov. 2016, *Vlasák Jr. 1611/5-J* (Holotype in PRM, isotype in JV and BJFC).

##### Etymology.

*Gilbertsonii* (Lat.): in honor of Prof. R.L. Gilbertson, the American mycologist.

##### Fruitbody.

Basidiocarps annual, resupinate, difficult to separate from the substrate, corky when dry, up to 10 cm long, 8 cm wide and 0.8 mm thick at center. Pore surface pale buff to buff when dry; sterile margin indistinct, very narrow to almost lacking; pores round to angular, 2–3 per mm; dissepiments thick, entire. Subiculum cream, corky, thin, about 0.3 mm thick. Tubes light buff, corky, about 0.5 mm long.

##### Hyphal structure.

Hyphal system dimitic: generative hyphae bearing clamp connections, hyaline, thin-walled; skeletal hyphae dominant, thick-walled, frequently branched, IKI–, CB–, tissues unchanging in KOH.

##### Subiculum.

Generative hyphae infrequent, hyaline, thin-walled, occasionally branched, 2–3 µm in diam; skeletal hyphae dominant, hyaline, distinctly thick-walled, frequently branched, interwoven, 1.5–3 µm in diam.

##### Tubes.

Generative hyphae infrequent, hyaline, thin-walled, occasionally branched, 1–3 µm in diam; skeletal hyphae dominant, distinctly thick-walled, frequently branched, interwoven, 2–4 µm in diam. Cystidia absent; cystidioles present, fusiform, hyaline, thin-walled, 13–23 × 4.5–6 µm. Basidia pear-shaped to barrel-shaped with 4-sterigmata and a basal clamp connection, occasionally with a few large guttules, 21–25 × 10–14 µm; basidioles dominant, similar in shape to basidia, but slightly smaller. Dendrohyphidia absent. Some irregular-shaped crystals present among tube tramal structures.

##### Spores.

Basidiospores oblong, hyaline, thick-walled, with tuberculate ornamentation, IKI–, CB+, 12–15(–16) × (5.5–)6–8 µm, L = 14.07 µm, W = 6.9 µm, Q = 1.83–2.15 (n = 60/2).

##### Additional specimen examined (paratype).

USA. Arizona, Chiricahua Mt., Turkey Canyon, on dead tree of *Quercus*, 5 Sep. 2012, *Vlasák Jr. 1209/63-J* (JV, dupl. in BJFC).

#### Haploporus microsporus

Taxon classificationFungiPlagiorchiidaHaploporidae

Meng Zhou&Y.C.Dai sp. nov.

MB829585

Figs 8 , 9

##### Diagnosis.

Diﬀers from other *Haploporus* species by the combination of a resupinate habit, a dimitic hyphal structure with dextrinoid skeletal hyphae, distinct small pores, 7–9 per mm, the presence of dendrohyphidia, and distinct small ellipsoid basidiospores measuring 5.3–6.7 × 3–4.1 µm.

**Figure 8.**
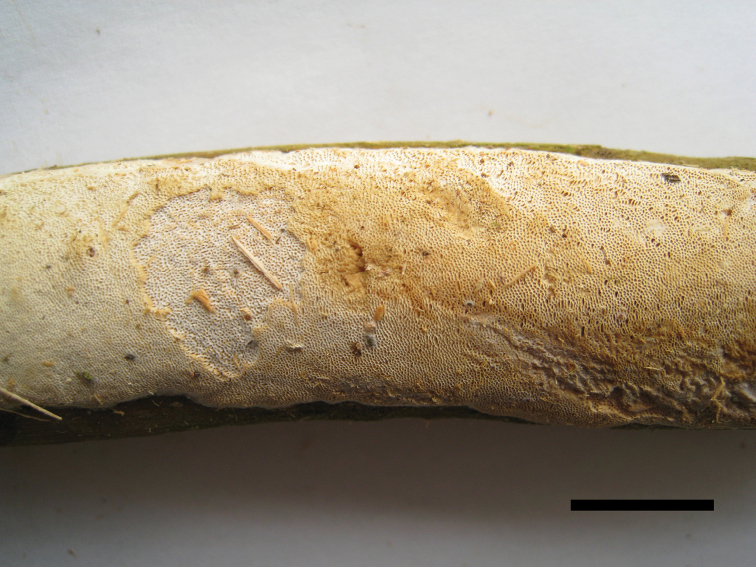
A basidiocarp of *Haploporusmicrosporus* (Holotype). Scale bar: 1.0 cm.

**Figure 9.**
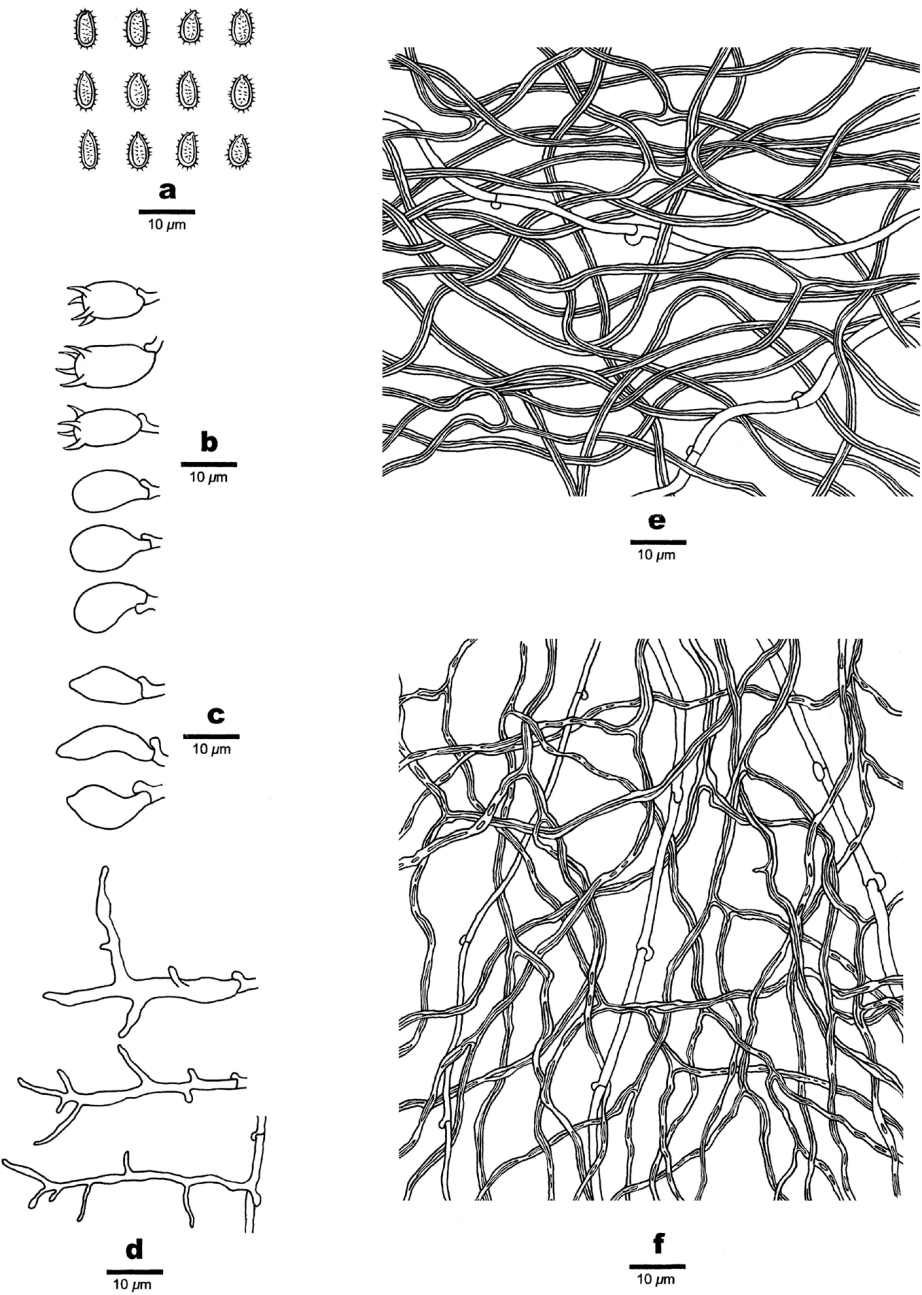
Microscopic structures of *Haploporusmicrosporus* (Holotype). **a** Basidiospores **b** Basidia and Basidioles **c** Cystidioles **d** Dendrohyphidia **e** Hyphae from subiculum **f** Hyphae from trama.

##### Holotype.

CHINA. Hainan Prov., Ledong County, Jianfengling Nat. Res., on dead angiosperm tree, 23 March 2011, *Dai 12147* (Holotype in BJFC).

##### Etymology.

*Microsporus* (Lat.): referring to the small basidiospores of this species.

##### Fruitbody.

Basidiocarps annual, resupinate, adnate, soft corky when fresh, become corky upon drying, odor- or tasteless when fresh, up to 20 cm long, 4.5 cm wide and 2 mm thick at center. Pore surface pinkish buff to clay-buff when dry; sterile margin indistinct, very narrow to almost lacking; pores angular, 7–9 per mm; dissepiments thick, entire. Subiculum cream, corky, thin, about 0.2 mm thick. Tubes light buff, corky, about 1.8 mm long.

##### Hyphal structure.

Hyphal system dimitic: generative hyphae bearing clamp connections, hyaline, thin-walled; skeletal hyphae dominant, thick-walled, frequently branched, dextrinoid, CB–, skeletal hyphae swollen in KOH.

##### Subiculum.

Generative hyphae infrequent, hyaline, thin-walled, rarely branched, 1.5–2.5 µm in diam; skeletal hyphae dominant, hyaline, thick-walled with a narrow to wide lumen, frequently branched, interwoven, 1.5–3 µm in diam.

##### Tubes.

Generative hyphae infrequent, hyaline, thin-walled, rarely branched, 1.5–3 µm in diam; skeletal hyphae distinctly thick-walled with a narrow lumen to subsolid, frequently branched, interwoven, 1–2 µm in diam. Cystidioles present, fusiform, 10–20 × 3.5–6 µm. Basidia barrel-shaped with 4-sterigmata and a basal clamp connection, 11–16 × 5.5–6.5 µm; basidioles dominant, similar in shape to basidia, but slightly smaller. Dendrohyphidia abundant, frequently branched. Some irregular-shaped crystals present among tube tramal structures

##### Spores.

Basidiospores ellipsoid, hyaline, thick-walled, with tuberculate ornamentation, dextrinoid, CB+, 5.3–6.7(–7) × (2.9–)3–4.1 µm, L = 5.98 µm, W = 3.90 µm, Q = 1.78 (n = 30/1).

#### Haploporus pirongia

Taxon classificationFungiPlagiorchiidaHaploporidae

(G. Cunn.) Meng Zhou, Y.C.Dai&T.W. May comb. nov.

MB829650

Figs 10 , 11

Poria pirongia G. Cunn., Bull. N.Z. Dept. Sci. Industr. Res., Pl. Dis. Div. 72: 39 (1947) (Basionym)

##### Etymology.

the epithet *pirongia*, derived from the type locality, Mount Pirongia, is a noun in apposition, and therefore remains spelt the same when transferred from *Poria* to *Haploporus*, despite the latter genus being masculine in gender.

##### Fruitbody.

Basidiocarps annual, resupinate, difficult to separate from the substrate, soft corky when fresh, corky upon drying, odor- or tasteless when fresh, up to 8 cm long, 2 cm wide and 1.7 mm thick at center. Pore surface white to cream when fresh, pale brownish when bruised, pinkish buff to clay-buff upon drying; sterile margin very narrow to almost lacking; pores round to angular, 3–4 per mm; dissepiments thick, entire. Subiculum cream, corky, thin, about 0.3 mm thick. Tubes light buff, corky, about 1.4 mm long.

##### Hyphal structure.

Hyphal system trimitic: generative hyphae bearing clamp connections, hyaline, thin-walled, frequently branched; skeletal hyphae dominant, thick-walled to subsolid, hyaline to slightly yellowish, frequently branched; binding hyphae abundant, slightly thick-walled, IKI–, CB+, tissues unchanging in KOH.

##### Subiculum.

Generative hyphae frequent, hyaline, thin-walled, frequently branched, 2.3–3.5 µm in diam; skeletal hyphae dominant, hyaline, distinctly thick-walled with a narrow lumen to subsolid, occasionally branched, interwoven, 2.5–4 µm in diam; binding hyphae abundant, slightly thick-walled,1–2 µm in diam.

##### Tubes.

Generative hyphae frequent, hyaline, thin-walled, frequently branched, 1.7–3.5 µm in diam; skeletal hyphae distinctly thick-walled with a narrow to wide lumen, frequently branched, interwoven, 2.5–4 µm in diam; binding hyphae slightly thick-walled,1–2.5 µm in diam. Cystidia absent; cystidioles present, fusiform, occasionally with an apical simple septum, sometimes with a few small guttules, 21–28 × 5–7 µm. Basidioles dominant, similar in shape to basidia, but slightly smaller, occasionally with a few large guttules; basidia pear-shaped to barrel-shaped with 4-sterigmata and a basal clamp connection, 21–35 × 8–11 µm. Hyphae at dissepiment usually thick-walled with simple septum. Dendrohyphidia absent. Some irregular-shaped crystals present among tube tramal structures.

##### Spores.

Basidiospores oblong-ellipsoid to cylindrical, hyaline, thick-walled, with tuberculate ornamentations, some with a guttule, IKI–, CB+, 11–14(–15) × (4.8–)5.2–7 µm, L = 12.35 µm, W = 6.11 µm, Q = 1.83–2.15 (n = 90/3).

##### Specimens examined.

AUSTRALIA. Victoria, Melbourne, Dandenong Ranges Botanical Garden, on dead branch of *Rhododendron*, 12 May 2018, *Dai 18659*, *18660* & *18661* (MEL, dupl. in BJFC); on dead branch of *Eucalyptus*, 12 May 2018, *Dai 18662* (MEL, dupl. in BJFC). NEW ZEALAND. Omahu Bush, on *Melicytus*, 15 Feb 2010, Cooper (PDD 95714, dupl. in BJFC).

**Figure 10.**
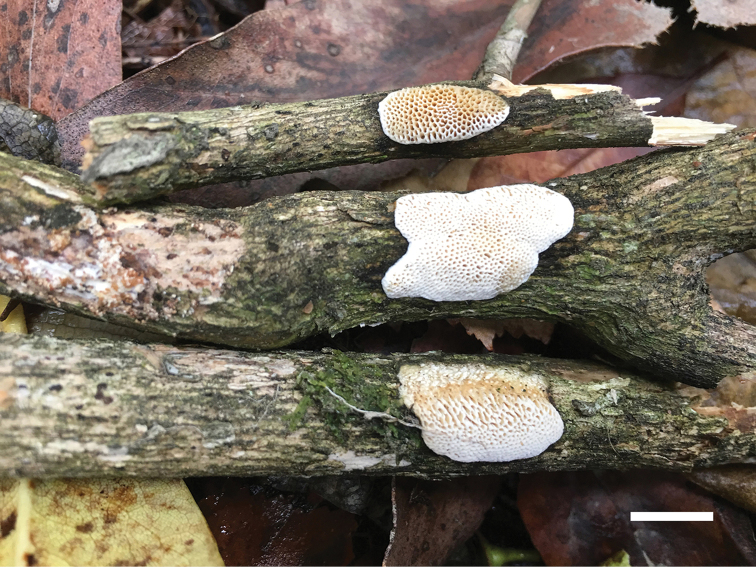
Basidiocarps of *Haploporuspirongia*. Scale bar: 1.0 cm.

**Figure 11.**
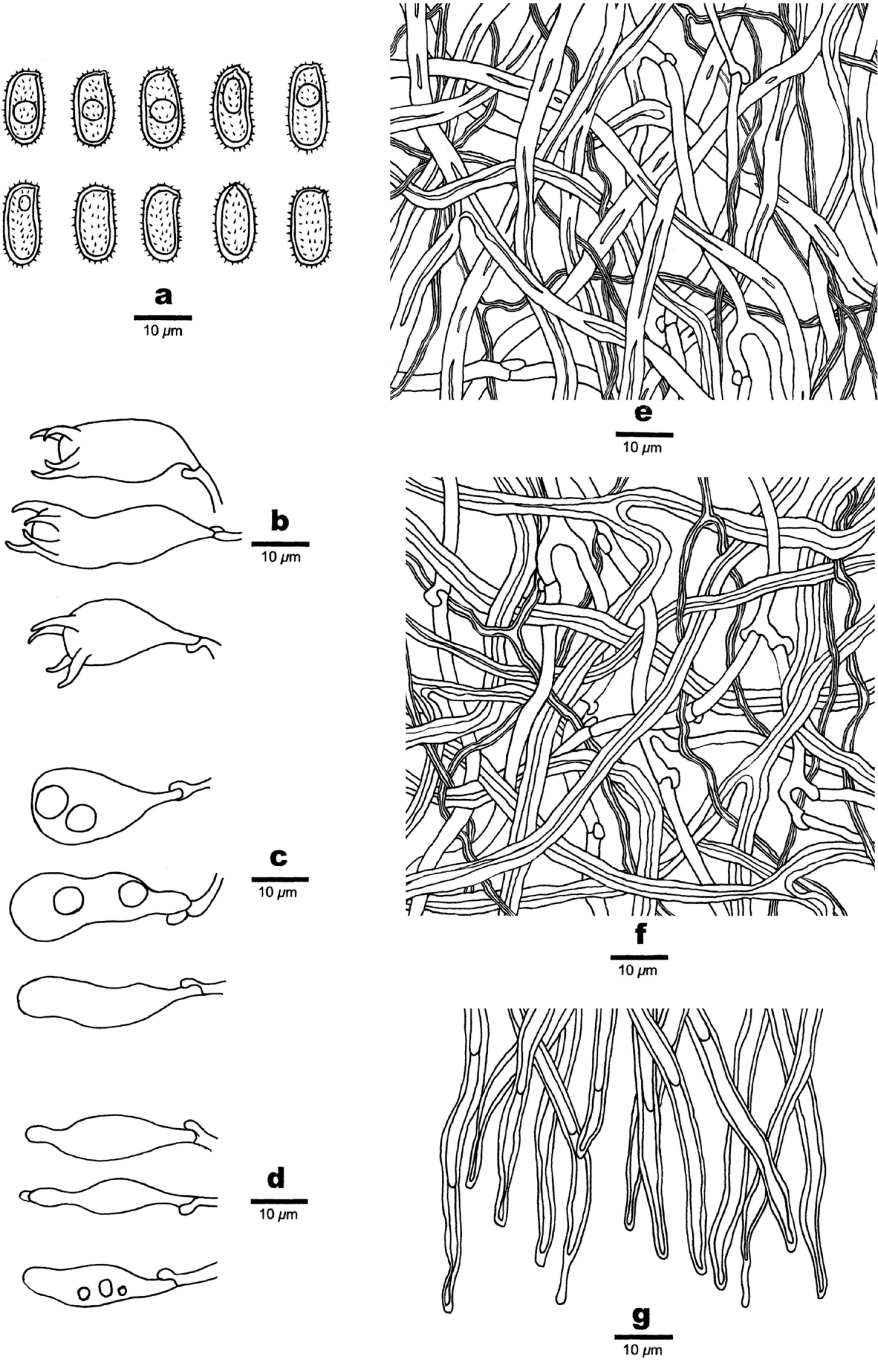
Microscopic structures of *Haploporuspirongia*. **a** Basidiospores **b** Basidia **c** Basidioles **d** Cystidioles **e** Hyphae from subiculum **f** Hyphae from trama **g** Hyphae at dissepiment.

#### Haploporus odorus

Taxon classificationFungiPlagiorchiidaHaploporidae

(Sommerf.) Bondartsev & Singer in Singer, Mycologia 36: 68 (1944)

=Haploporusamarus X.L. Zeng & Y.P. Bai, Acta Mycol. Sin. 12(1): 13 (1993). Holotype: China, Jilin Province, Northeast Normal University, Changchun, NENU, Zeng 1931.

##### Notes.

*Haploporusamarus* was described from NE China (Zeng and Bai 1993). The type was studied, and its morphology is in agreement with that of *H.odorus*.

#### Haploporus subtrameteus

Taxon classificationFungiPlagiorchiidaHaploporidae

(Pilát) Y.C.Dai&Niemelä, in Dai, Niemelä and Kinnunen, Ann. bot. fenn. 39(3): 181 (2002)

=Pachykytosporawasseri Zmitr., Malysheva & Spirin, *Ukrainskiy Botanichnyi Zhurnal* 64(1): 42 (2007) Holotypus: Russia, Samara Reg., Stavropol Dist., Zhiguli Nat. Res., Padus avium, 12.09.2006, V.F. Malysheva, E.F. Malysheva, I.V. Zmitrovich, isotypus, LE 214872.

##### Notes.

In our phylogenies (Figs 1 and 2), *P.wasseri* (Zmitrovich et al. 2007) nested within *H.subtrameteus* clade. In addition, there are not major morphological differences between the two taxa (Zmitrovich et al. 2007).

## Discussion

In the ITS-based phylogeny (Fig. 2), *Haploporusangustisporus* is closely related to *H.alabamae and H.nanosporus*. Morphologically, *Haploporusangustisporus* may be confused with *H.alabamae* in having approximately the same basidiospores size (9.5–12.5 × 4–5.5 µm vs. 10–13.5 × 4–5 µm) but *H.alabamae* has a trimitic hyphal system and lacks cystidioles (Gilbertson and Ryvarden 1986–1987). *Haploporusnanosporus* differs from *H.angustisporus* by its smaller pores (9–12 per mm vs. 3–5 per mm), non-dextrinoid skeletal hyphae, and smaller basidiospores (5–6 × 3–4 µm vs. 10–13.5 × 4–5 µm, Piątek 2005).

*Haploporusgilbertsonii* is closely related to *H.cylindrosporus*, *H.thindii*, *H.nepalensis* and *H.tuberculosus*. However, *Haploporusthindii* differs from *H.gilbertsonii* by its distinctly slimmer basidia (20–37 × 6.5–9.1 µm vs. 21–25 × 10–14 µm) and the absence of cystidioles (Yu et al. 2005). *Haploporusnepalensis* is distinguished by its smaller basidiospores (5.5–11.5 × 4.5–6.5 µm vs. 12–15 × 6–8 µm) and the absence of cystidioles (Piątek 2003). Whereas *Haploporustuberculosus* is distinguished from *H.gilbertsonii* by its trimitic hyphal system and longer basidia (30–43 × 11–13.5 µm vs. 21–25 × 10–14 µm, Ryvarden and Gilbertson 1994).

The *Haploporusnanosporus* and *H.microsporus* clades are sister clades and *Haploporusnanosporus* is closely related to *H.alabamae* and *H.angustisporus*. *Haploporus* and *H.nanosporus* both have small basidiospores and occurs in tropical ecosystems,and all other differing in having larger basidiospores. However, *H.nanosporus* differs from *H.microsporus* by the absence of dendrohyphidia at the dissepiments, a trimitic hyphal system and absence of cystidioles (Piątek 2005). In addition, *Haploporusalabamae* differs from *H.microsporus* through a trimitic hyphal system and absence of cystidioles (Gilbertson and Ryvarden 1986–1987). *Haploporusangustisporus* differs from *H.microsporus* by its longer basidiospores (10–13.5 × 4–5 µm vs. 5.3–6.7 × 3–4.1 µm).

In the ITS-LSU based phylogeny (Fig. 1), *Haploporuscrassus* is closely related to *H.papyraceus* and *H.subpapyraceus*. However, morphologically *Haploporuspapyraceus* differs from *H.crassus* by the presence of dendrohyphidia at the dissepiments, absence of cystidioles and thin-walled basidioles (Ryvarden and Johansen 1980). *Haploporussubpapyraceus* also differs from *H.crassus* in having dextrinoid skeletal hyphae and thin-walled basidioles (Shen et al. 2016).

*Haploporuspirongia* is related to *H.odorus*, but the latter has a perennial and pileate basidiocarp with strong anise odor, ovoid basidiospores and lacks cystidioles (Niemelä 1971). *Haploporuspirongia* resembles *H.thindii* and *H.subpapyraceus* by sharing resupinate basidiocarps with approximately the same pore size. However, *Haploporusthindii* has a dimitic hyphal structure, lacks cystidioles, and has a distribution in subtropical India and valley of Tibet of China (Natarajan and Kolandavelu 1993, Dai et al. 2007). Moreover, *H.subpapyraceus* has ellipsoid basidiospores (9–12 × 5.5–8 μm, Shen et al. 2016).

Gilbertson and Ryvarden (1987) reported *Haploporustuberculosus* (as *Pachykytosporatuberculosa*) from the USA, but only in a small region of southern Arizona where it should be “quite common on oaks, especially in Chiricahua Mountains”. Locally, we have collected in this region only *H.gilbertsonii* and believe that, in most cases, this species was mistaken for *H.tuberculosus* in Arizona. The presence of *H.tuberculosus* in America is questionable.

## Supplementary Material

XML Treatment for Haploporus angustisporus

XML Treatment for Haploporus crassus

XML Treatment for Haploporus gilbertsonii

XML Treatment for Haploporus microsporus

XML Treatment for Haploporus pirongia

XML Treatment for Haploporus odorus

XML Treatment for Haploporus subtrameteus
